# Dispositional empathy as a driver of inter-individual neural phase synchrony

**DOI:** 10.1038/s41598-025-01485-2

**Published:** 2025-05-14

**Authors:** Mari Falcon, Silja Martikainen, Valtteri Wikström, Tommi Makkonen, Katri Saarikivi

**Affiliations:** 1 Cognitive Brain Research Unit, Faculty of Medicine, University of Helsinki, P.O. Box 21, 00014 Helsinki, Finland; 2 Faculty of Arts, University of Helsinki, P.O. Box 4, 00014 Helsinki, Finland; 3 Faculty of Educational Sciences, University of Helsinki, P.O. Box 4, 00014 Helsinki, Finland; 4 Department of Psychology, Faculty of Medicine, University of Helsinki, PO Box 21, Helsinki, Finland

**Keywords:** Inter-brain, Hyper-scanning, Synchrony, Empathy, Collaboration, EEG, Cooperation, Social behaviour, Empathy

## Abstract

In social neuroscience inter-individual neural phase synchrony has become a widely studied phenomenon, and has been linked to a variety of social outcomes. However, the cognitive processes underlying the emergence of this synchrony remain largely unknown. In this study, we used a two-person face-to-face collaborative task to investigate the potential of dispositional empathy—the general tendency of an individual to imagine and experience the feelings and experiences of others—as a driver of inter-individual neural synchrony during collaboration. Electroencephalography from 46 participants was used to examine phase synchrony, measured as circular correlation coefficients, between the two interacting individuals’ brain signals. We found significant inter-brain synchrony in the high alpha, beta, and gamma frequency bands. This synchrony was particularly prominent in the inter-brain connectivity measured between central regions and a range of other regions. Furthermore, a specific dimension of dispositional empathy, namely the collaborators’ tendency to transpose themselves imaginatively into the feelings and actions of others, predicted the level of synchrony in the beta and gamma frequency bands. Hence, we demonstrate that dispositional empathy plays a significant role in the emergence of inter-individual neural phase synchrony.

## Introduction

Multi-person brain activity recording during natural interaction has become a widely used method in social neuroscience in the last decades. This method, also referred to as hyperscanning, has enabled investigating different inter-personal dynamics of neural patterns^1–3^. Inter-individual neural synchrony, meaning simultaneous changes in the brain’s electrical or hemodynamic activity between two or more individuals, has been found to automatically emerge during social interaction^4–9^. This synchrony has been connected to several positive social phenomena during face-to-face^7,10–12^ and recently also online^13–16^ interaction. However, it is unclear which cognitive mechanisms underlie the emergence of this synchrony^17^. Common individual processing of social and non-social stimuli is understood to play a part in the synchrony. Certain socio-cognitive processes, such as mutual engagement and joint attention^7,12,15,18^ and the brain’s empathy mechanisms^19,20^ have also been proposed.

Only recently, broader theories for explaining the role and underlying processes of inter-brain synchrony have been presented. The Mutual Prediction Theory presented by Kingsbury et al.^19^ posits that synchronization arises from individuals simultaneously maintaining representations of similar cognitive states during interaction. Specifically, Kingsbury et al. propose that two distinct brain systems are engaged in an individual’s brain during social interaction: one that governs the individual’s own cognition and behavior, and another that predicts the cognition and behavior of others. The similarity in neural activity across these systems, as measured in multiple brains, results in coherence between the overall brain activity of the interacting individuals. Shamay-Tsoory et al. introduce a Social alignment model suggesting that three levels of social alignment - a motor system, emotional contagion, and conformity - are highly connected. The interaction and integration of these levels lead to both behavioral and neural synchronization among individuals^20^. Building on this model, Gvirts et al.^21^ propose the Mutual Social Attention System. This theory suggests that synchronization between what they describe as the “mutual social attention systems” of interacting individuals - primarily involving the temporoparietal junctions and prefrontal cortices - enhances participants’ ability to focus on the interaction, its participants, and its objectives. By fostering such synchronization, this system promotes social alignment, ultimately supporting successful and rewarding social interactions.

As interpreting, predicting, and reacting in accordance with the mental states of others form the core of the proposed theories, empathy mechanisms can be expected to have a crucial role in the emergence of inter-brain synchrony. Empathy-driven synchrony could reflect higher-level processing of the mental states and intentions of others, also referred to as Cognitive Empathy^19^, or more implicit empathy processes, such as neural resonance and emotional contagion, often referred to as Affective Empathy^20^. The general level of an individual’s tendencies to employ processes related to Cognitive and/or Affective Empathy is often referred to as trait or dispositional empathy.

The majority of hyperscanning studies do not include measures of dispositional empathy as potential predictors of inter-brain synchrony. There is also considerable variation in the methods used for both recording brain activity as well as calculating neural synchrony. Studies investigating similarity in slower changes in neural patterns between individuals have linked inter-brain synchrony to the level of dispositional empathy^22^. However, a significant portion of today’s hyperscanning studies focus on more specific temporal phase synchrony between individuals. It is unclear whether the emergence of this type of phase synchrony reflects empathic processes.

While some studies investigating inter-individual phase synchrony have explored situational empathy measures along with synchrony, the role of different dimensions of dispositional empathy as predictors of synchrony remains underexplored. Oftentimes the use of dispositional empathy measures has been limited to controlling for group differences, instead of investigating the relationship between inter-brain synchrony and empathy^23–25^. Only few studies have considered dispositional empathy as a driver of inter-brain phase synchrony. In a natural classroom setting, Dikker et al. found that Empathic Distress (a specific dimension of Affective Empathy measured using the Interpersonal Reactivity Index questionnaire), predicted student-to-group phase synchrony^12^. Reinero et al. on the other hand reported an absence of a significant relationship between dispositional Cognitive Empathy and group-level inter-brain phase synchrony. These findings suggest that specific types of empathic processes may contribute to inter-individual synchrony^26^. However, these studies could offer a limited amount of control due to the natural classroom setting, involving varying interaction between the multiple group members. In both studies, trait empathy was also quantified using only a single scale and synchrony was measured as an average of synchrony occurring at different frequencies of neural oscillations. In order to identify the underlying links between dispositional empathy and inter-brain phase synchrony, this calls for a comprehensive exploration of the role of different dimensions of dispositional empathy in modulating synchrony of neural oscillations measured at varying frequencies.

In the current study, we investigated the relationship between inter-brain phase synchrony and dispositional empathy, utilizing Electroencephalography (EEG) and different measures of Cognitive and Affective Empathy. The collaborative setting included a problem-solving task (part 1) followed by a natural real-life sales interaction (part 2) between two individuals (a lawyer and a potential client). In this paper we present the results from the first part of the study. The rationale for investigating collaboration during a problem-solving task was to create quantifiable data on the flow and success of the dyad’s collaborative problem-solving during a standardized situation that could be comparable between the dyads. We first identified socially relevant synchrony, ie. synchrony arising specifically due to cognitive processes related to interaction during the problem-solving task instead of spurious synchrony. This was done by comparing the measured inter-brain synchrony values with synchrony values calculated between time-shuffled permutations of the interacting participants’ EEG data. We then explored the associations between the socially relevant synchrony and the different social and cognitive measures, such as dispositional Cognitive Empathy, dispositional Affective Empathy, general reasoning skills and the collaborative outcome. Previously, inter-brain phase synchrony in the alpha, beta and gamma frequency bands has been associated with social phenomena such as joint attention^14,27^, social gaze^10,11^, level of cooperation^4,13,28,29^, coordinated actions^5,23^, social affiliation^10^, and collaborative performance^15^. Based on these findings, we expected to find socially relevant inter-individual neural phase synchrony in the mentioned frequency bands. We also assumed that different social and cognitive measures would predict partly distinct types of synchrony found in either different frequency bands and/or measured between different electrode sites. Additionally, we expected the level of neural synchrony between individuals to vary as a reflection of the level of collaborative success in the collaborative reasoning task.

## Methods

### Participants

46 participants took part in the study as part of a dyad, formed of one lawyer and one potential client. The lawyers were recruited from a Finnish mid-size law firm, and the clients were recruited through the law firm’s list of potential business clients and social media channels. Due to the limited number of lawyers in the company, 10 lawyers took part in the study twice, and were assigned a new potential client each time. This resulted in 28 dyads. To keep the level of familiarity constant, the dyads were randomly formed of participants who were unfamiliar with one another prior to the study.

All participants were healthy volunteers with no diagnosed neuropsychological disorders or visual/hearing impairments. All participants reported identifying as male or female. Among lawyers 58.8% were male and 41.2% female. Among clients 60.7% were male and 39.3% female. The participants’ random assignment to dyads led to 11 same-gender and 17 mixed dyads. The mean age of lawyers was 42.8 years (Min=29, Max=57, SD=9.6 years). The mean age of clients was 48.4 years (Min=30, Max=59, SD=6.5 years). Among lawyers 94.1 % had a graduate degree and 5.9 % had an undergraduate degree. Among clients 82.1 % had a graduate degree, 7.1 % had an undergraduate degree, 7.1 % had a high school degree, and 3.6 % had a doctoral degree. The Informed consent was obtained from all participants. This included informed consent for publishing potentially identifiable images of participants in online open-access publications. The study was performed in accordance with the guidelines of the Declaration of Helsinki, and was approved by the Ethical Review Board in the Humanities and Social and Behavioural Sciences of the University of Helsinki.

### Protocol

Before the day of the experiment, both participants were sent two empathy questionnaires and an individual reasoning test to fill in online before the experiment. The experiment was held in a meeting room located on the premises of the law firm. To prevent contact beforehand, the participants were assigned a different arrival time. Upon arrival, the participants were accompanied by one of the researchers to separate rooms for preparations, during which EEG caps were placed. After this they were escorted into the experiment room, sat down facing each other around a table, and were asked to shortly introduce themselves. One of the researchers then instructed the participants on the collaborative reasoning task consisting of five different puzzles^30^. The researcher measured completion time and errors of each puzzle. To avoid potential effects of the actual success in the task on the perceived quality of collaboration, the results of the success in the task was not disclosed with the participants. During the experiment, two other researchers were operating the EEG recordings. The three researchers and two participants were present in the room throughout the experiment. During the task, video of each participant was recorded with cameras placed next to the experiment table, and Electrocardiogram (ECG) was recorded with small sensors placed on the upper torso of the participants. After the collaborative reasoning task was completed, the participants continued onto carry out the separate sales interaction, and a questionnaire concerning the perceived social outcomes of this interaction. Findings related to the sales interaction as well as the video and ECG data will be reported separately.

### The collaborative reasoning task and individual reasoning skills

**Fig. 1:**
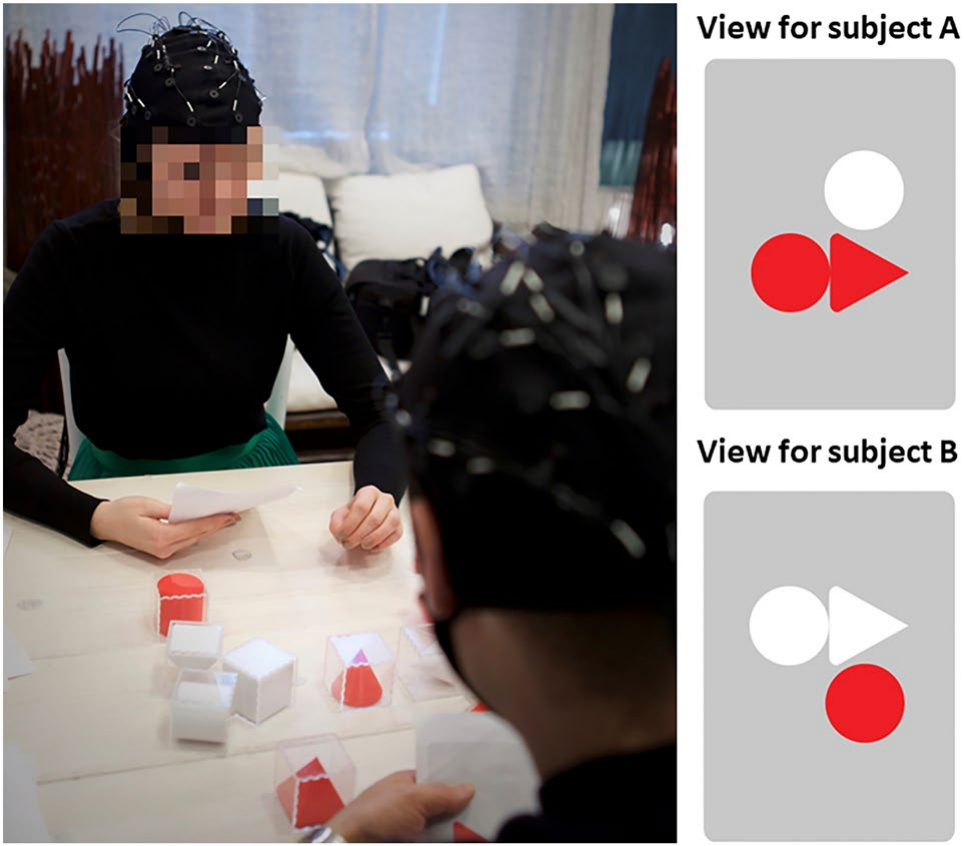
A demonstration of the collaborative task setting. An example of the views of the cards of the participants is presented on the right. Each participant was only able to access their own view. This required participants to communicate and share their perspectives, in order to settle upon a common solution for the 3D construction on the table.

To investigate collaboration, we used the CoBlok collaborative block design task^30,31^as it requires both participants’ contribution in the task and enables quantifying collaborative success. Also, because the roles of both collaborators in the task are symmetrical, it enabled minimizing potential differences caused by the natural roles of the participants as representatives of either the law firm or another company. Coblok consists of three-dimensional target puzzle configurations that can only be solved with collaborative effort using a variety of physical three-dimensional puzzle blocks. For each puzzle, the participants are each provided a card with a two-dimensional image of shapes, representing the same three-dimensional target block configuration from a specific viewing angle (as viewed either from any of the four sides or from above the configuration). The viewing angle is unique for each of the two participants, and only combining the views of the two angles enables building the three-dimensional target configuration. Participants must collaborate in building the accurate target configuration by combining the information of both participants’ card. Participants are not allowed to show their card to the other person, and must thus communicate the information they have, either verbally or by utilizing the physical puzzle blocks used for constructing the configuration. The participants must come to a mutual conclusion, in which they believe the three-dimensional configuration represents both participants’ card’s image from any given angle. Once a mutual conclusion has been reached, the puzzle is considered completed, and the accuracy of the configuration can be determined by the conductor of the task. If the three-dimensional configuration accurately represents each participant’s card from some angle, the configuration is correct. An example of the experimental setting and the two cards representing two different viewing angles of a specific configuration are presented in Fig. 1.

In this study, we used a time limit of 12 min for each puzzle. If the dyad failed to finish the puzzle within the time frame, the researcher would instruct the dyad to move on to the next task. In order to avoid repetition of the same task among the lawyers participating more than once, we used two different sets of puzzles (Set 1 and Set 2) in the study. These sets consisted of the same number of puzzles, with an equal number of blocks required for completing the puzzles. In both cases the number of blocks required increased as the task progressed^31^. Each dyad was randomly assigned to complete either Set 1 or Set 2, with the exception of dyads including a lawyer attending the study for the second time. In this case the dyad was assigned to complete the set that the lawyer had not completed during their first participation. The puzzles within each set were always completed in the same sequence to minimize bias from presentation order. For each correctly constructed puzzle, the dyad received 1 point. In case the dyad was unable to complete the puzzle in the given time frame, or settled upon an incorrect configuration, they would receive 0 points. The researcher conducting the task checked the accuracy of each finished puzzle based on images of 3-dimensional models of the configurations. These images were kept hidden from the participants. In case of any unclarities, the accuracy was verified by inspecting video recordings of the session. The combined score of all five puzzles was used in the analyses as the index of collaborative task performance. As there was a difference in the success rate for the puzzles in Set1 compared to Set2, the assigned task set was considered as a factor in the later stages of analyses along with considering whether the lawyer was taking part in the experiment for the first or second time. Due to the visuospatial nature of the task, we expected participants’ individual reasoning skills to affect the dyad’s performance in the task. We therefore used the abbreviated version of The Raven Standard Progressive Matrices to control for these skills^32,33^. The validity of the abbreviated version of the test has been reported as high (Cronbach’s alpha =0.80–0.83^33^). As task-specific neural synchrony is assumed to partially reflect shared individual processes during interaction, we expected that within-dyad similarity in individual reasoning processes may play a part in explaining inter-individual neural synchrony during the Coblok task. We therefore considered both the within-dyad mean as well as the within-dyad difference in the Raven’s standard progressive matrices scores in our analyses.

### Empathy measures

The Reading the Mind in the Eyes test (RME) was used as a measure of Cognitive Empathy skills^34^. More specifically, RME measures an individual’s ability to recognize emotions from the faces of strangers using only information gathered from an image of the person’s eyes. The scale consists of 36 items and the individual’s test score is based on the number of correct items. In addition, we used the Interpersonal reactivity index (IRI), a 28-item self-report questionnaire consisting of four subscales, with 7 items each^35^. Two of the IRI subscales, Fantasy (F) and Perspective Taking (PT), are considered to reflect tendencies related to Cognitive Empathy, while Empathic Concern (EC) and Personal Distress (PD) are considered to reflect tendencies related to Affective Empathy^35,36^. More specifically, the Perspective Taking subscale measures one’s tendency to spontaneously adopt the psychological point of view of others; the Fantasy subscale measures one’s tendencies to transpose themselves imaginatively into the feelings and actions of fictitious characters; the Empathic Concern subscale measures feelings of sympathy and concern for others; whereas the Personal Distress subscale measures feelings of personal anxiety and unease in tense interpersonal settings^35^. The internal consistency of the IRI has been reported as high, with Cronbach’s alpha coefficients for each subscale as follows PD: males, 0.77; females, 0.75; EC: males, 0.68; females, 0.73; PT: males, 0.71; females, 0.75; F: males, 0.78; females, 0.79^35^. In the original study, the average scores for females and males, respectively, were F: 18.75 and 15.73, F(1,1176) = 96.28, p < 0.001; PT: 17.96 and 16.78, F(1,1180) = 18.25, p < 0.001, EC: 21.67 and 19.04, F(1,1180) = 129.09, p < 0.001, and PD: 12.28 and 9.46, F(1,1181) = 103.10, p < 0.001^35^. The internal consistency for the RME has been reported as acceptable (Cronbach’s alpha .67), with an average score for males 26.28 (SD 3.24) and females 26.64 (SD 3.24)^37^ (However, see criticism of the validity of RME in the Limitations section).

In the analyses, we used the within-dyad means of the overall RME score, and the within-dyad mean of each IRI subscale score to assess the associations between inter-individual neural synchrony and different dimensions of empathy.

### EEG recording and preprocessing

During the experiment, EEG was recorded from both participants using two Neuroscan Synamps amplifiers and Neuroscan’s 32-channeled Quick-Caps, with a sampling rate of 512 Hz, and with Cz set as the reference channel. For measuring electrooculogram (EOG), one electrode was placed next to the lateral canthus of the eye, and one below the center of the eye. A custom-built trigger box was used to send triggers simultaneously to both participants’ EEG data enabling temporally matching the datasets. The preprocessing of the EEG data was carried out with Matlab R2022a (MathWorks Inc., USA) and EEGLAB 2021.1^38^. EEG data recorded from the starting point of the first puzzle until the end point of the last puzzle were included in the preprocessing and analyses phases. Channel locations were added using the standard Quick-Cap 10/20 layout based on the 32-channeled caps used in the study. Data were visually inspected for any highly noisy or flat channels. These channels were then excluded in the following steps. A high-pass filter was applied at 0.5 Hz (-6dB cutoff at 0.25 Hz), followed by the application of a low-pass filter at 48 Hz (-6dB cutoff at 49.056 Hz). Each dataset was then segmented into 3-second epochs for exclusion of segments with significant artefacts. This window was chosen to ensure reliable phase estimation in the following synchrony analyses. The threshold for automatic segment rejection was set to $\pm 500$ ${\mu \hbox {V}}$ and all EEG data were also visually inspected. As a result, two dyads’ datasets were excluded from all analyses due to the poor quality of one of the participant’s reference electrode data or insurmountable movement artefacts. Next, an Independent Component Analysis (ICA) was run for the data consisting of the remaining segments using all non-excluded EEG and EOG channels. EEGLAB’s IClabel tool^39^ was used for identifying ocular and muscle artefacts. For the ocular artefacts, any artefacts flagged by IClabel as ocular with an $\ge 80 \%$ probability were selected for visual inspection and removed unless the properties did not match those of an ocular artefact. For muscle artefacts, we used a threshold of $\ge 70 \%$ probability, as there is more individual variance in their properties^40,41^. After this, the channels excluded from the previous preprocessing steps, were interpolated. The data was then re-referenced to a common average. Finally, the EEG data was temporally matched between the two participants of each dyad. All segments previously removed from one of the participant’s EEG data were also removed from the dyad’s other participant’s EEG data. As the time spent on each task as well as the number of removed segments varied per dyad, the dyads’ final datasets consisted of a minimum of 87 segments (261 s) and a maximum of 497 (1491 s) segments [mean = 244.54 (733 s), SD = 102.08 (306.24 s)].

### Inter-brain connectivity analysis

Based on previous research on the robustness of different phase synchrony indices used in hyperscanning studies, we chose the circular correlation coefficient (CCorr)^42^. CCorr measures the covariation of phase variation between two signals at a specific time point, relative to the mean covariation over time (ie. relative to the level of covariation expected at random between two signals). For calculating the CCorr between the two participant’s EEG, we used the Hyperscanning Python Pipeline (HyPyP) developed specifically for inter-brain analyses^43^. The EEG data included in the analysis was decomposed into four frequency bands of interest; lower alpha (7.5–10 Hz), higher alpha (10.5–13 Hz), beta (13.5–29.5 Hz) and gamma (30–48 Hz). The frequency bands were defined based on previously discovered associations between these frequency bands and different social outcomes^4,5,7,10,13,15,23,27–29,44^. Although synchrony in the theta band has also been linked with social phenomena, it is assumed to largely reflect synchrony between neural activity related to observed speech and auditory processes^8,45^. Since these processes were neither controlled for nor of specific interest in the current study, the theta band was excluded from the analyses.

CCorr values were first calculated as the average of the absolute CCorr values of all 3-second segments between each of the two participants’ channel combinations for each frequency band separately. This 3-second window allowed for reliable phase estimation by resulting in a minimum of approximately 20 cycles in each frequency band. In order to limit the number of statistical tests and consider expected spatial properties of socially significant synchrony, six regions of interest (ROIs) were formed: right frontal, left frontal, right central, left central, right parietal, and left parietal. The selection of ROIs matches prior and recent studies of inter-brain synchronization^5,10,46,47^. The occipital and temporal regions were excluded to reduce findings of spurious synchrony related to visual and auditory processing which varied dyad-to-dyad in this study.

**Fig. 2:**
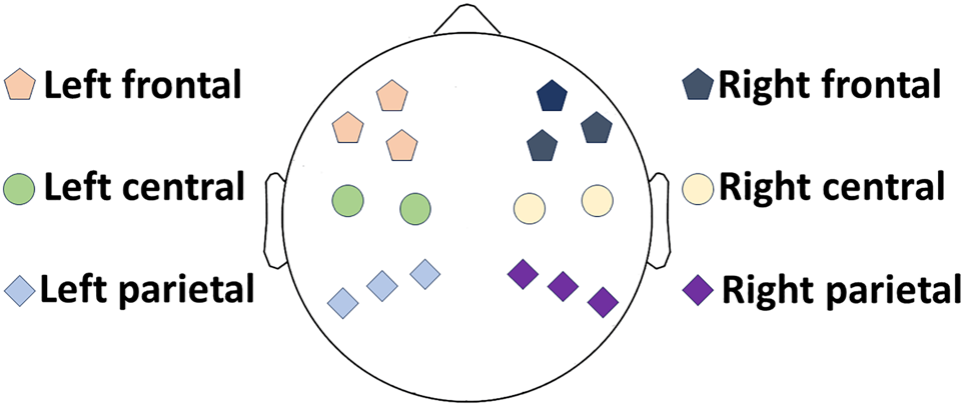
Electrode locations for each region of interest.

The synchrony for each region-region combination was then calculated as the mean of the synchrony values between all channels included in the two regions. This was done separately for each frequency band, resulting in 36 synchrony indices, representing the mean synchrony over time in different frequency bands between the different regions of the interacting individuals. The layout of the channels included in each region is presented in Figure 2. It is worth noting that as no structural imaging, and hence no source localization was carried out, the regions/ROIs refer to the locations of the specified groups of electrode sites. The regions therefore represent approximate locations of brain activity measured over certain areas of the scalp, and not precise brain regions.

To identify synchrony emerging as a result of interaction, we controlled for spurious synchrony by utilizing a permutation method used widely in hyperscanning studies^48–50^. We wanted to ensure that the inspected synchrony was specific to the actual temporal timing of the cognitive states of the individuals, and not merely synchrony occurring between the two participants’ EEG signals at any differing time points. We therefore compared the synchrony values calculated for the temporally matched EEG signals to those calculated for each dyad’s temporally shuffled segments of the signals at random time points. We calculated the region-frequency CCorr values between 300 time-shuffled combinations of the data segments of each dyad’s data. We then averaged each CCorr value over the 300 permutations. This resulted in the same number of ($6^2$) connectivity indices as for the temporally matched data.

### Statistical analyses

To assess whether there are systematic differences between the group of lawyers and clients concerning any of the used psychometric measures, we conducted Independent Samples T-tests for each of the variables. The results of the T tests are presented in Table 1. As no significant differences were found between the lawyers compared to clients, no differentiation was made between the two groups in later stages of analyses.

**Table 1:** The minimum, maximum, mean and standard deviation of the participants’ score in each empathy scale (Interpersonal Reactivity Index subscales and the Reading the Mind in the Eyes scale) and score in the individual reasoning task (Raven standard progressive matrices) separately for lawyers and potential clients. T and p values of the T tests comparing lawyers’ and potential clients’ scores are presented on the right side of the table. No significant differences were found between lawyers and clients concerning any of the scores.

Role	N	Mean	SD	T	p
Individual reasoning skills					
Lawyer	16	6.33	1.63	0.165	0.870
Client	26	6.25	1.55	–	–
RME score					
Lawyer	16	27.40	3.81	1.379	0.176
Client	26	26.00	2.73	–	–
IRI fantasy					
Lawyer	16	24.00	4.42	$-0.296$	0.769
Client	26	24.52	5.88	–	–
IRI perspective taking					
Lawyer	16	26.00	3.65	0.727	0.472
Client	26	25.12	3.75	–	–
IRI personal distress					
Lawyer	16	16.47	3.93	1.670	0.103
Client	26	14.08	4.62	–	–
IRI empathic concern					
Lawyer	16	25.80	3.88	0.195	0.846
Client	26	25.48	5.57	–	–

**Fig. 3:**
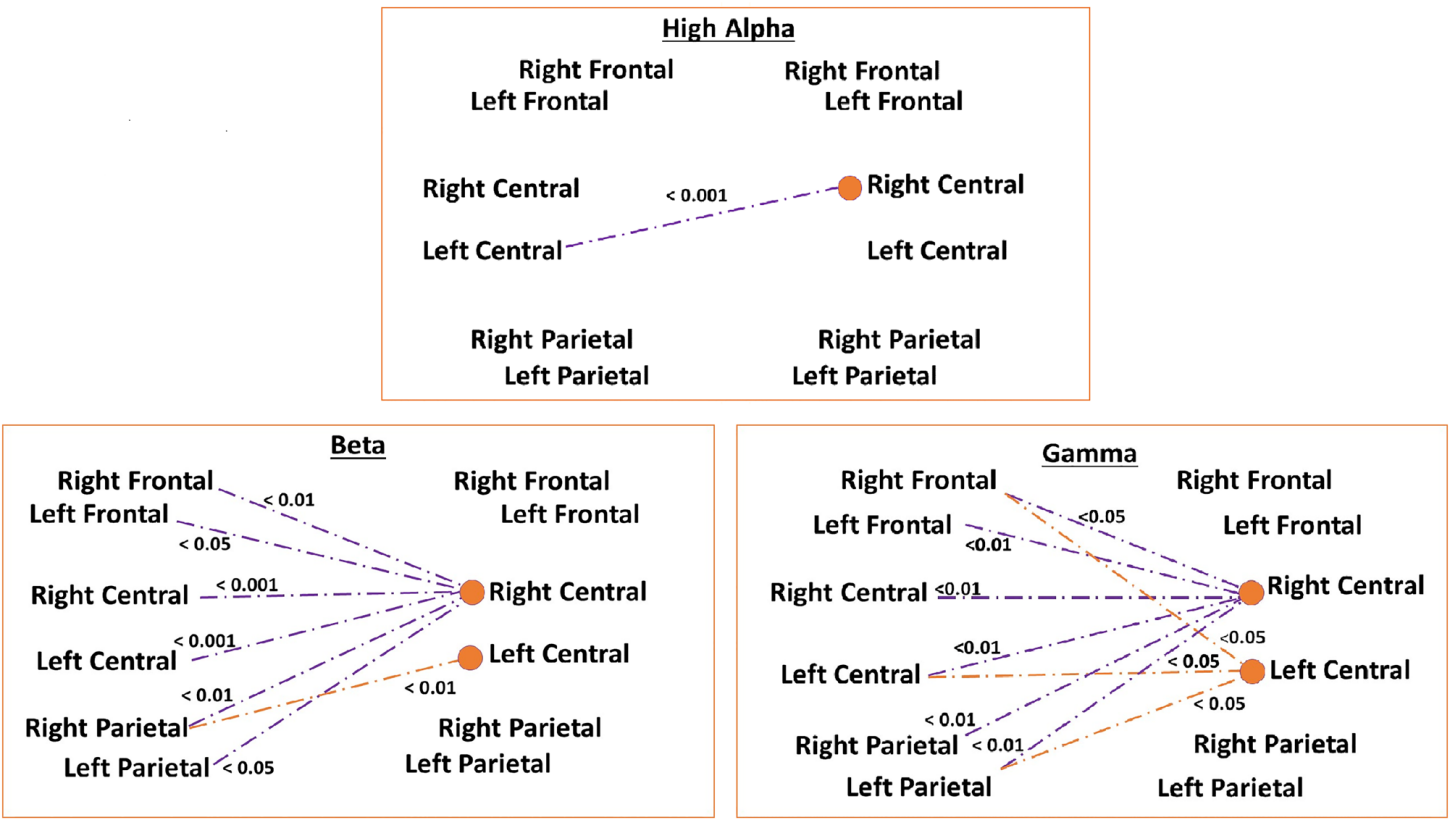
The significant synchrony between the brain regions of two interacting individuals. The values in the three images represent the Bonferroni adjusted p values of paired samples Wilcoxon’s tests comparing the 26 dyads’ synchrony values during the task to the synchrony values of the dyads’ surrogate data. The orange circles on the right represent the clustering of these connections. In the images, the brain regions are duplicated into two sets of the same regions (on the left and right side of each image) for clarity—the synchrony indices used in the analyses are in fact the grand means of the synchrony both from participant A to B and from participant B to A, meaning these visualized synchrony connections represent two-way synchrony between the two interacting individuals.

As assumptions of parametric tests were not met regarding all frequency band-region combinations, we used a Paired Wilcoxon’s test to compare the synchrony values calculated between the temporally matched datasets, to the synchrony values calculated between the surrogate datasets. In order to avoid type 1 errors, we used Bonferroni correction to assess the significance of the synchrony found between the collaborating participants’ temporally matched EEG signals. The synchrony values found significant after the multiple comparison correction (adjusted p-values < 0.05), were selected for further investigation. In the case of multiple neighboring regions sharing significant synchrony with the same region of the other participant, these synchrony values were clustered into one synchrony index. A visualization of the final clustered synchrony indices can be found in Fig. 3.

Multiple linear regression analyses were conducted to test whether the collaborative outcome and trait empathy (measured by RME and the four IRI subscales) were associated with significant neural synchrony. To examine these associations in all frequency bands between the different regions, we ran five separate regression models. Each model predicted one clustered synchrony value. Due to the explorative nature of the study concerning the five different empathy dimensions and the collaborative outcome, the inclusion of these predictors was carried out using a stepwise method. These predictors were entered into the model one at a time, based on significance thresholds of p<0.05 for entry and p>0.10 for removal.

All models were adjusted for the following potential covariates (all covariates were included simultaneously in the models): (1) time spent on interaction, (2) within dyad mean score of individual reasoning skills, (3) within dyad difference in individual reasoning skills, (4) whether the participant was attending for the first or the second time, (5) the task set used, (6) the interaction of first or second attendance and the task set. The significance of the regressors was assessed based on false discovery rate (FDR) adjusted p-values of < 0.05. Potential multicollinearity between all variables was assessed, with a minimum tolerance of > 0.2.

In addition to including time spent on interaction as a predictor in the models, we used Pearson correlation coefficients together with visual inspection to assess the association between time and inter-individual synchrony. Previous research suggests there may be a systematic negative effect of time on inter-individual neural synchrony, whether explained by weakened EEG signals over time resulting from technical factors (and hence decline in potential to detect synchrony) or factors related to interaction itself^15,26^. To investigate the effect of time on the current data, we examined the change in the CCorr values over all time points, ie. every valid 3-second segment.

The assumption of homoscedasticity of variance was met for all five models, and no concerns regarding multicollinearity between any of the variables included in the models arose (VIF < 5 and tolerance > 0.2).

## Results

### Synchrony emerging during interaction

Significant synchrony was found in the high alpha, beta, and gamma frequency bands during the collaborative task. Figure 3 represents a visualization of the synchrony in each frequency band clustered based on spatial distribution of the synchrony values. The lines represent the connectivity found statistically significant as a result of the Paired Wilcoxon test, with the Bonferroni adjusted p values presented in the image for each connection. In all three frequency bands, connectivity between the central regions and various other regions accounts for a large portion of the neural synchrony arising during interaction. No significant synchrony was found in the lower alpha band.

#### Synchrony in the high alpha frequency band

In the high alpha frequency band, synchrony emerged specifically between the right central and left central regions with a CCorr value of 0.131 (Min = 0.123, Max = 0.149, SD = 0.005). The equivalent CCorr value for the time-shuffled data was 0.127 (Min = 0.125, Max = 0.128, SD = 0.0006).

#### Synchrony in the beta frequency band

In the beta band, two different synchrony clusters were identified: (1) connectivity of the right central region with the right frontal, left frontal, right central, left central, right parietal, and left parietal regions, (2) connectivity of the left central region with the right parietal region. The mean CCorr value for the cluster reflecting the connectivity of the right central region with other regions was 0.072 (Min = 0.063, Max = 0.109, SD= 0.011) and the mean CCorr value for the cluster representing the connectivity of the left central region with other regions was 0.067 (Min = 0.062, Max = 0.076, SD = 0.003). The CCorr values for the time-shuffled data were 0.065 (Min = 0.063 , Max = 0.067, SD = 0.0006) and 0.064 (Min = 0.064, Max = 0.067, SD = 0.0005) respectively.

#### Synchrony in the gamma frequency band

Two different synchrony clusters were also identified in the gamma band: (1) connectivity of the right central region with the right frontal, left frontal, right central, left central, right parietal and left parietal region, (2) connectivity of the left central region with the right frontal, left central, and left parietal regions. The mean CCorr value for the cluster reflecting the connectivity of the right central region with other regions was 0.076 (Min = 0.061, Max = 0.135, SD= 0.017) and the mean CCorr value for the cluster representing the connectivity of the left central region with other regions was 0.065 (Min = 0.059, Max = 0.080, SD = 0.005). The CCorr values for the time-shuffled data were 0.062 (Min = 0.061, Max = 0.062, SD = 0.0003) and 0.062 (Min = 0.061, Max = 0.062, SD = 0.0002) respectively.

### Synchrony over time

As expected, the inspection of the changes in level of inter-individual neural synchrony over time revealed a slight negative trend in the association between time spent on interaction and synchrony in all frequency bands for all region-to-region connections (R <0 between all synchrony values and time). However, the correlation was only statistically significant concerning the right central region’s connectivity with other regions in the beta band (R $= -0.029$, p = 0.023) and in the gamma band (R = $-0,073$, p < 0.001). In the regression models, task completion time was not found to predict the level of inter-brain synchrony in any of the frequency bands (see Table 2).

### Synchrony, collaborative performance and individual reasoning skills

Collaborative performance was not associated with any of the synchrony indices in any of the frequency bands. The regression analysis revealed a significant negative association between the within-dyad mean score of individual reasoning skills and inter-individual neural synchrony in the beta band measured over the left central and right parietal region (B $= -$ 0.002 , SE 0.0007, p $=$ 0.007), as well as in the gamma band measured over the left central region and the cluster of other regions (B $= -0.003$ , SE 0.0012, p $=$ 0.019). The within-dyad difference in the individual reasoning score was also found to predict beta synchrony between the left central and right parietal regions (B $= -0.001$ , SE 0.0004, p $=$ 0.003), as well as gamma synchrony both between the right central region and the cluster of other regions (B $= -0.006$ , SE 0.0029, p = 0.040), and between the left central region and the cluster of other regions(B $= -0.002$ , SE 0.0007, p $=$ 0.012) (see Table 2).

### Synchrony and trait empathy

No significant associations were found between inter-individual neural synchrony and Affective Empathy, measured as the scores of the IRI Empathic concern and Personal distress subscales. Also, neither the RME score nor the IRI Perspective-taking subscales were found to predict synchrony. A higher within-dyad mean score of the IRI Fantasy subscale, on the other hand, was associated with higher inter-brain synchrony in the beta and gamma frequency bands. Before the FDR correction this association was found for all socially relevant synchrony in the beta and gamma bands. After the FDR correction, this effect remained significant concerning synchrony between the left central and right parietal region in the beta band (B = 0.0008 , SE 0.0001, p = 0.001), and between the left central region and the cluster of other regions in the gamma band (B = 0.0009 , SE 0.0003, p = 0.039) (See Table 2).

### Assessment of the regression models

When considering the overall model significance, only two of the regression models were found statistically significant. These were the models predicting beta synchrony between the left central and right parietal region ($R^2$ = 0.713, p = 0.003) and gamma synchrony between the left central region and cluster of other regions ($R^2$ = 0.607, p = 0.025). As mentioned, the assumption of homoscedasticity of variance was met for all five models, and no signs of multicollinearity between any of the variables included in the models were found (VIF < 5 and tolerance > 0.2). Hence, the issues related to the overall model powers may indicate an insufficient sample size and/or that the combination of variables included did not adequately explain the variance in all synchrony indices. Also, while the stepwise regression method was used to enable the explorative approach of this study, the potential risk of overfitting when using stepwise methods should be considered especially in relatively small samples. Table 2 summarizes the results for each model, indicating the continuous variables entered and their corresponding significance levels. The used task set or first versus second participation of the lawyer were not found to predict synchrony in any of the frequency bands (p > 0.05 in each model). The within-dyad mean IRI Fantasy score consistently demonstrated significance across multiple models, suggesting its robust predictive power in the context of the current experimental setting.

**Table 2:** The continuous predictors initially included in the five regression models predicting the mean level of socially relevant inter-brain synchrony between the interacting individuals. In the subheadings, RC stands for right central, LC stands for left central, and RP stands for right parietal regions. The p values represent the adjusted values, with significant p values ( p < 0.05 ) after FDR correction marked in bold.

Predictor	$\beta$	SE ($\beta$)	T	*p*	CI (95%)
High alpha RC-LC					
Raven mean	$-0.00056$	0.00165	$-0.338$	0.739	$-0.00406, 0.00294$
Raven difference	$-0.00049$	0.00099	$-0.491$	0.630	$-0.00259, -0.00161$
Time spent on interaction	$-0.00001$	0.00001	$-0.070$	0.945	0.00001, 0.00001
Beta RC cluster					
Raven mean	$-0.00586$	0.00290	$-2.020$	0.062	$-0.01204, 0.00032$
Raven difference	$-0.00358$	0.00175	$-2.050$	0.058	$-0.00731, 0.00014$
Time spent on interaction	$-0.00037$	0.00002	$-1.585$	0.134	$-0.00009, 0.00001$
IRI fantasy	0.00151	0.00065	2.312	0.208	0.00012, 0.00289
Beta LC-RP					
Raven mean	$-0.00216$	0.00068	$-3.150$	**0.007**	$-0.00362, -0.00070$
Raven difference	$-0.00149$	0.00041	$-3.611$	**0.003**	$-0.00237, -0.00061$
Time spent on interaction	$-0.00001$	0.00001	$-1.796$	0.093	$-0.00002, 0.00000$
IRI fantasy	0.00079	0.00015	5.156	**0.001**	0.00046, 0.00047
Gamma RC cluster					
Raven mean	$-0.00996$	0.00474	$-2.101$	0.053	$-0.02006, 0.00014$
Raven difference	$-0.00642$	0.00285	$-2.250$	**0.040**	$-0.01250, -0.00034$
Time spent on interaction	$-0.00006$	0.00004	$-1.642$	0.121	$-0.00015, 0.00002$
IRI fantasy	0.00250	0.00106	2.353	0.196	0.00024, 0.00477
Gamma LC cluster					
Raven mean	$-0.00318$	0.00120	$-2.638$	**0.019**	$-0.00575, -0.00061$
Raven difference	$-0.00208$	0.00073	$-2.869$	**0.012**	$-0.00363, -0.00054$
Time spent on interaction	$-0.00002$	0.00001	$-2.084$	0.055	$-0.00004, 0.00000$
IRI fantasy	0.00085	0.00027	3.161	**0.039**	0.00028, 0.00143

## Discussion

In the current study we explored multiple dimensions of dispositional empathy as potential predictors of inter-individual neural phase synchrony. To this aim, we used a natural face-to-face social setting involving two participants completing a collaborative task, while EEG was simultaneously measured. As expected, inter-individual neural synchrony arising during interaction was found in the high alpha, beta and gamma frequency bands. In all frequency bands, specifically connectivity of the central brain regions (both right and left) with the other regions of interest, was found to account for the synchrony arising during social interaction.

Cognitive, but not Affective, trait empathy was found to predict the level of synchrony in the beta and gamma frequency bands. Specifically the within-dyad mean of the IRI Fantasy score, representing the individual’s tendencies to transpose oneself imaginatively into the feelings and actions of fictitious characters, was positively associated with inter-brain synchrony in the beta and gamma bands. As successful completion of the collaborative task required listening to the partner describe their vantage point of the construction and to imagine the target from their point of view, we expect that the nature of the task required empathic functions driven less by implicit, and rather by explicit cognitive processing. This may partially explain the divergent findings compared to previous inter-brain research investigating group synchrony in which Affective empathy - namely the personal distress dimension of the IRI scale - was found to predict synchrony^12^. The natural group setting in the previous study may be more likely to elicit spontaneous contagion of emotions while the Coblok task used in the current study requires deliberately forming similar cognitive representations of the puzzle constructions. Not only might the current task particularly activate processes related to Cognitive (rather than Affective) Empathy, it may specifically hinder more automatic empathic responses by shifting focus away from the emotional cues of the other. However, differences in the over-all study design and analyses approaches (such as specifying between different frequency bands versus examining the average synchrony across different frequency bands) do not permit direct comparisons between these studies, and continued systematic research in different contexts is needed.

Contrary to our expectations and findings in previous studies^7,15^, no significant relationship was found between collaborative performance and inter-individual neural synchrony in any of the frequency bands. As expected, there was a slight negative trend between synchrony and time in all frequency bands. There was also variation in the time it took for dyads to complete each puzzle. Although the effect of time was controlled for in the analyses, this caused certain additional considerations. As the scoring system originally created for the collaborative task is highly dependent on time^31^, we used a simplified scoring system to explore the association between synchrony and collaborative success. It is possible that a standard time spent on interaction across all dyads, and the ability to use the original more detailed scoring system, would have enabled revealing an association between synchrony and task performance.

Higher within-dyad mean of individual reasoning skills was associated with lower synchrony in the beta and gamma bands. This could reflect the need for compensating for lower reasoning skills by employing other cognitive processes that promote completing the task. These may include processes such as those related to joint attention and/or empathy which are then reflected as increased inter-individual neural synchrony. The within-dyad difference in individual reasoning skills was also found to predict synchrony in the beta and gamma bands, with a smaller difference (ie. higher similarity) in reasoning skills associated with higher levels of synchrony. This finding is in line with our hypothesis, and could mean that inter-brain synchrony partly reflects similarities in cognitive processes related to performing the specific task.

Activity in the alpha band has generally been associated with sensory stimulation, memory and attentional processes^51^, as well as various socio-cognitive functions^52–54^. Activity in the lower alpha band has been associated with attentional processes, whereas higher alpha has been linked with imitation and observation of movement. Furthermore, higher alpha activity reflecting observation and prediction of motor actions has been linked to the right hemisphere, whereas activity reflecting imitation has been linked with the left hemisphere^55^. In hyperscanning studies, inter-brain phase synchrony in the alpha band has been associated with spontaneous motor imitation^5^, actively coordinated actions^23^, speech^44^, and joint attention^15,27^. Alpha synchrony over the central regions has also been found to increase during eye contact^11^. Our current findings are in line with previous research proposing a significant role of inter-brain synchrony over the right central region in the higher alpha band during social interaction. Synchrony found specifically between the right central and left central regions could reflect the interplay of motor prediction and control between the other and oneself as a manifestation of for example naturally occurring social alignment.

Modulation in the beta band has previously been found especially during individuals carrying out motor tasks or tasks that require sensorimotor integration^56^. Hyperscanning findings suggest that increased inter-individual beta synchrony over the motor cortex improves synchronization of movements among individuals^7^. Although alignment of movements was not required for task completion in our experiment, the natural face-to-face setting allowed for intrinsic social behavior to occur spontaneously. This means beta synchrony measured over the premotor and motor cortex could partly reflect implicit synchrony of motor actions. Additionally, beta synchrony has been found to increase during cooperative compared to non-cooperative settings involving competition^13^ or defection^4^. The CoBlok task relies heavily on cooperation between the interacting individuals, as a competitive or deceitful approach would be detrimental for finding a common solution. Therefore, our finding related to beta synchrony arising during the task provides support for previous findings linking beta synchrony specifically to cooperation. The association between beta synchrony and the IRI Fantasy dimension of empathy found in our study suggests a significant role of the tendency to adapt the point of view of others in the emergence of neural synchrony during collaboration.

At an individual level, the role of gamma activity is seen to reflect especially memory and spatially widely distributed integrative functions within the brain^57,58^. This may be reflected in previous hyperscanning findings concerning gamma phase synchrony connected to partly similar social functions as alpha and beta synchrony, such as social gaze^10^ and cooperation^13,28,29^. Gamma synchrony has also been linked to the level of social affiliation between the interacting individuals^10^. In our study, significant gamma synchrony occurred between strangers, and was not seen to increase during the course of time on the group level. Our finding concerning the link between the IRI Fantasy scale and gamma synchrony might suggest a role of this specific empathy dimension as a potential mediator between synchrony and social affiliation. In future work, measuring the level of social affiliation and synchrony between initial strangers before, during, and after interaction in groups with high versus low empathic traits could shed light on the associations between empathy, social affiliation and inter-brain synchrony.

Considering previous findings discussed above, an association between Affective Empathy and inter-brain synchrony, especially in the alpha and beta bands, might have been expected. No such association was however found in our study. It is possible that while utilization of brain activity recording techniques with high temporal precision, such as EEG, are sufficient for revealing processing related to Cognitive Empathy, better spatial precision would be required for unraveling inter-individual patterns of more implicit processes. As using EEG limits the measured signals to the outer cortex, this also hinders the chance of findings reflecting synchrony of activity in subcortical regions, which are crucial for certain functions related to Affective Empathy. However, the limited previous findings related to dispositional empathy and inter-brain phase synchrony have linked synchrony specifically with the level of Affective Empathy in natural group settings using EEG^12^.

This together with our current findings highlights the relevance of the nature of the task at hand when it comes to the relationship between inter-brain synchrony and empathy. The association between synchrony and the specific Fantasy dimension of empathy was most significant over the left central region’s inter-brain connectivity with the right parietal region in the beta band, and the left central region’s inter-brain connectivity with the right frontal, left central, and left parietal regions in the gamma band. This suggests an important role of inter-brain connectivity of the left central region with other brain regions when it comes to the type of synchrony reflecting the mentioned empathic tendencies.

### Limitations

We would like to highlight that the absence of precise source localization limits the accuracy of spatial estimates for EEG signals measured over the different regions. We therefore recommend using these findings as a foundation for future research employing improved spatial localization techniques. Additionally, due to our technical setup, we were unable to match behavioral or audio data with the EEG data at a desired temporal level. Being able to utilize speech and motor information would have enabled inspecting the potential link between empathy-related motor actions (such as alignment of movements) and synchrony. This would have also allowed investigating synchrony over especially the temporal regions, which have previously been found relevant for inferring others’ mental states based on social cues. Future research should combine movement and speech data with neural data in order to extend the understanding of the link between neural synchrony and empathic expressions and tendencies.

Another limitation of the current study was the absence of an active control condition. A control condition in which interaction is minimized, and/or that corresponds to the used condition but does not require similar cognitive processing as completing the block design task, would enable drawing further conclusions about the role of inter-brain synchrony. Such control conditions would also be useful for further eliminating potential effects of the task-related stimuli on synchrony. However, as the task used in the current study did not require simultaneous movements and the processed task-related stimuli was non-identical among the two individuals (each card held unique information), we consider the preprocessing and analysis steps sufficient for identifying socially relevant synchrony.

Recently, some concerns related to the psychometric properties of the RME scale have been raised, warranting caution when using the RME scale as a measure of Cognitive Empathy^50,59,60^. In our study an association was found between inter-brain synchrony and the IRI Fantasy subscale, while no association was found between inter-brain synchrony and the RME scale. As the Fantasy subscale is described to measure fairly similar processes as the RME scale, our findings support the concerns regarding the validity of RME as a measure of Cognitive Empathy. Future hyperscanning studies could benefit from using scales that provide a more generalized measure of dispositional empathy (for example the Empathic Experience Scale^61^). Also, particularly given the relatively small sample size (N = 28 dyads) in this study, we acknowledge that utilizing a stepwise approach and including several covariates in the regression models may increase the risk of overfitting. While we controlled for multiple comparisons using FDR correction, carefully selected the covariates, and tested for multicollinearity, future studies with larger sample sizes and the use of regularization techniques may provide additional confidence in the robustness of these findings.

It is also worth noting that as the Coblok task requires communication between the interacting participants, it evokes many cognitive processes leading to different inter-individual social dynamics. In addition to empathy processes, different combinations of for example personality traits and/or communication styles may lead to shared or differing states of cognitive processing. This in turn could result in higher or lower levels of inter-individual neural synchrony. Additionally, other cognitive functions such as attentional and memory processing may play a key role in explaining phase synchrony in different frequency bands and should be considered in future work. In this study all participants were also exposed to the same sequence of puzzles within the Coblok task. While this fixed-order task design was used to ensure consistency, it may also introduce a potential order effect.

Finally, the fact that our study consisted of lawyers and management-level professionals, should be taken into account when assessing the generalizability of the results. As no significant differences were found between lawyers and potential clients concerning the studied psychometric measures, the two groups were treated as equal. This, however, does not rule out the potential influence of the different roles on the studied interaction. Despite the equal role of the two participants during the Coblok task, it is possible that certain systematic social dynamics between the lawyers and the potential clients prevail. While the aim of the current study was to assess the potential role of trait empathy as a driver of inter-individual synchrony, future work will benefit from further considering different inter-personal dynamics during the interaction in addition to the aforementioned individual social attributes.

### Conclusions

Through our current work, we have demonstrated that dispositional empathy plays an important role in the emergence of inter-brain phase synchrony, and suggest measures of empathy skills and tendencies should be included more widely in hyperscanning studies. Given our current findings, compared to previous research, we propose that the task used in the experimental setting modulates the relationship between empathy and inter-brain synchrony, with specific empathic processes accounting for synchrony depending on the cognitive requirements of the task at hand. In line with recent suggestions^17^, we also encourage combining bodily information with neural activity and considering active minimally social control settings in future work.

## Data Availability

The datasets generated during the current study are not publicly available as explicit consent for sharing the data has not been given by the participants. Anonymized datasets are, however, available from the corresponding author upon reasonable request with the condition of participants’ consent.
